# Responsive Regulation of Energy Transfer in Lanthanide‐Doped Nanomaterials Dispersed in Chiral Nematic Structure

**DOI:** 10.1002/advs.202303235

**Published:** 2023-07-28

**Authors:** Yuxia Luo, Qingdi Liu, Ping He, Liang Li, Zhao Zhang, Xinping Li, Guochen Bao, Ka‐Leung Wong, Peter A. Tanner, Lijun Jiang

**Affiliations:** ^1^ College of Bioresources Chemical and Materials Engineering Shaanxi University of Science and Technology Xi'an Shaanxi 710021 China; ^2^ School of Life Sciences Central China Normal University Wuhan 430079 China; ^3^ Institute for Biomedical Materials and Devices (IBMD) Faculty of Science University of Technology Sydney Sydney NSW 2007 Australia; ^4^ Department of Chemistry Hong Kong Baptist University 224 Waterloo Road Kowloon Hong Kong SAR 999077 China

**Keywords:** cellulose nanocrystals, chiral nematic structures, energy transfer, lanthanides

## Abstract

The responsive control of energy transfer (ET) plays a key role in the broad applications of lanthanide‐doped nanomaterials. Photonic crystals (PCs) are excellent materials for ET regulation. Among the numerous materials that can be used to fabricate PCs, chiral nematic liquid crystals are highly attractive due to their good photoelectric responsiveness and biocompatibility. Here, the mechanisms of ET and the photonic effect of chiral nematic structures on ET are introduced; the regulation methods of chiral nematic structures and the resulting changes in ET of lanthanide‐doped nanomaterials are highlighted; and the challenges and promising opportunities for ET in chiral nematic structures are discussed.

## Graphical abstract

The responsive control of energy transfer (ET) plays a key role in the applications of lanthanide‐doped nanomaterials. Herein, the mechanisms of ET and the photonic effect of chiral nematic structures on ET are introduced; the regulation methods of chiral nematic structures and the resulting changes in ET of lanthanide‐doped nanomaterials are highlighted; and the challenges and promising opportunities for ET in chiral nematic structures are also discussed.

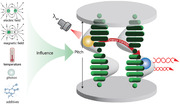

## 1 Introduction

Tripositive lanthanide ions (Ln^3+^) are critical resources due to their unique luminescent properties, including characteristic sharp emission bands, tunable visible‐near infrared emission wavelengths, large excitation–emission shifts, long lifetimes, and excellent photostability.^[^ ^1^ ^]^ These properties boost applications of Ln^3+^‐based materials from lighting to deep‐tissue imaging, biosensing,^[^ ^2^ ^]^ velocity sensing,^[^ ^3^ ^]^ nanothermometry,^[^ ^4^ ^]^ molecular structural change sensing,^[^ ^5^ ^]^ and stress sensing, ^[^ ^6^ ^]^ where energy transfer (ET) involving Ln^3+^ plays a key role. ET refers to the phenomenon where energy from an excited donor is nonradiatively transferred to an acceptor via a certain interaction mechanism. Upconversion and downshifting may both involve ET.^[^ ^7^ ^]^ Upconversion is a process where photons are emitted with higher energy than the incident radiation. A classic example is the near‐infrared excitation of Yb^3+^ to give red, green, or ultraviolet emission from Er^3+^. Downshifting comprises the emission of a (redshifted) photon with lower energy than absorbed. Ln^3+^ are characterized by narrow absorption cross sections resulting in weak emissions. Many strategies have been attempted to overcome this inherent limitation, including small‐molecule sensitization, host‐lattice modulation, core‐shell engineering, energy transfer or migration manipulation, photonic structure formation, plasmon resonance enhancement, and microlens amplification.^[^ ^7^ ^]^

**Figure 1:**
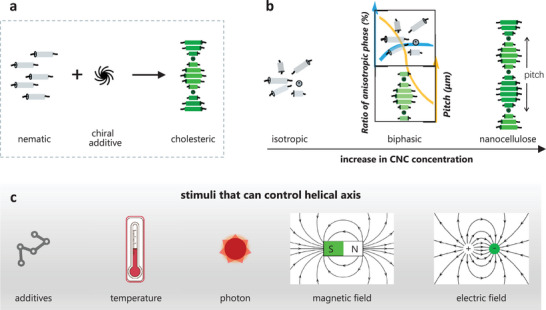
Schematic representation showing the self‐assembling processes of a) nematic liquid crystals with chiral additives and b) CNCs for the preparation of N*LCs. c) Stimuli that can control the pitch (distance for the director to rotate one full turn) of the cholesteric helix. Reproduced with permission.^[^ ^19^ ^]^ Copyright 2018, Wiley‐VCH.

Photonic crystals (PCs) are suitable materials to regulate light emission. Light propagation in specific directions for a given range of frequency in PCs is not allowed, due to their periodical variation in the local density of optical modes. This property of PCs can be exploited to control the spontaneous emission,^[^ ^8^, ^9^ ^]^ emission intensity, and lifetime of light emitters.^[^ ^10^ ^]^ Among the numerous materials that can be used to fabricate PCs, one promising type of material is a chiral nematic liquid crystal (N*LC). N*LCs have unique optical properties, for example, optical rotation and circular dichroism, making them excellent matrices to present tunable circularly polarized luminescence (CPL). N*LCs are prepared either by adding the chiral small molecule into the achiral nematic liquid crystal, or by assembling cellulose nanocrystals (CNCs) (**Figure** **1**). The emerging applications of N*LCs have extended to areas beyond color filters, flexible reflective displays, mirrorless lasers, smart sensors,^[^ ^11^ ^]^ UV shielding,^[^ ^12^ ^]^ foldable electronics,^[^ ^13^ ^]^ gratings,^[^ ^14^ ^]^ retroreflectors,^[^ ^15^ ^]^ CPL induction,^[^ ^16^ ^]^ nonlinear optics,^[^ ^17^ ^]^ optical metamaterials, and chiral nanophotonics.^[^ ^18^ ^]^ Thus, novel properties and innovative functionality are expected by encapsulating Ln^3+^‐doped nanoparticles (NPs) in N*LCs.

Given the already extensive overview of strategies to improve ET performance of Ln^3+^‐activated nanocrystals and their applications, we focus on recent advances in ET between Ln^3+^‐activated NPs, particularly in the chiral nematic structure. The review starts with a brief introduction of mechanisms and efficiencies of ET, followed by an extensive discussion of the formation and control of the chiral nematic structure. Then, the ET performance of Ln^3+^‐activated NPs in a chiral nematic structure is highlighted, which includes the preparation of luminescent NPs with a chiral nematic structure, and the responsive control of ET between Ln^3+^‐activated NPs therein. Challenges and prospective opportunities are also discussed at the end of this review.

## 2 ET in Ln^3+^‐Doped NPs

### 2.1 Spontaneous Emission Rate

For a donor‐acceptor pair, the excited donor has several ways to release energy, such as inner relaxation (vibrational and perhaps also rotational) to a lower electronic state; spontaneous emission of the donor (radiative decay); and ET from the donor to an acceptor, with migration (and sometimes cross‐relaxation) involving the same types of donors. The spontaneous emission process competes with ET to the acceptor.^[^ ^20^ ^]^ The rate of spontaneous emission is stimulated by vacuum fluctuations of the electromagnetic field. The rate is therefore determined by the particular electronic transition, the local density of states (LDOS), and the strength of the electromagnetic field (local field) at the position of the donor. In regard to the luminescence manipulation by the local field, we have summarized various models employed for the local field correction.^[^ ^21^ ^]^ According to the Fermi Golden Rule, the spontaneous emission rate of the donor (*k*) is proportional to the LDOS.

(1) $$k\;=\frac{4\pi^{2}}{h}\;|V{|}^{2}\rho \left(E\right)$$

Where *h* is the Planck constant, *V* is the zero‐point Rabi matrix element, and ρ(*E*) is the density of final states per unit of energy.^[^ ^22^ ^]^

### 2.2 ET Rate

ET is a process occurring from a donor to an acceptor and it has different forms and mechanisms. The forms can be categorized into radiative and nonradiative transfer, depending on whether real photons are emitted in the form of radiation. Radiative transfer refers to the transfer assisted by photons emitted by the donor. According to the interaction between donor and acceptor, nonradiative transfer is usually divided into multipolar and exchange ET, which have been well studied and widely exploited in many research works. Electric dipole‐electric dipole (ED‐ED) ET was elaborated by Dexter.^[^ ^23^ ^]^ It arises from a coulombic dipole‐dipole interaction and its rate varies as *R* ^−6^, where *R* is the distance between donor and acceptor. The donor releases its excited state energy and relaxes to a lower state nonradiatively; meanwhile, the acceptor absorbs the energy and populates its excited state. The ED‐ED ET rate (*k_ET_*) is given by:

(2) $$k_{\mathrm{ET}}=\frac{Q_{A}}{\tau_{Dr}}\;\frac{9h^{4}c^{4}}{128\pi^{5}}\frac{n^{-4}}{R^{6}}{\left\{\frac{E_{macr}}{E_{loc}}\right\}}^{4}\kappa^{2}J\left(E\right)$$

where *Q_A_* is the acceptor‐integrated absorption cross‐section; τ_Dr_ is the donor radiative lifetime; *c* is the speed of light; *n* is the refractive index; κ^2^ is the orientation factor of the donor and the acceptor with values between 0 and 4 with an average value 2/3 for an isotropic medium; the electric field term in curly brackets shows the macroscopic field and local field; and *J*(*E*) is the spectral overlap integral given as:^[^ ^24^ ^]^

(3) $$J\left(E\right)\;=\int_{0}^{\infty }f_{D}\left(E\right)f_{A}\left(E\right)E^{-4}\;dE$$

where *f* denotes oscillator strength, *E* is energy. Dexter has given formulae for higher‐order multipolar mechanisms.^[^ ^23^ ^]^ The exchange mechanism involves overlap between the donor and acceptor orbitals and the rate is given by:

(4) $$k_{ET}=\frac{4\pi^{2}}{h}\;Z^{2}J\left(E\right)$$

where *Z* ^2^ is a quantity that cannot be directly related to optical measurements, and which varies approximately in an exponential manner with −*R*.

In the case of radiative ET,^[^ ^25^ ^]^ the donor emits real photons which are then reabsorbed by the acceptor within a photon travel distance. The ET rate can be expressed as:

(5) $$k_{ET}=\frac{Q_{A}}{4\pi R^{2}\tau_{D}}\;J\left(E\right)$$

where τ_*D*_ is the lifetime of the donor.

Nonradiative and radiative ET can be differentiated by changes in lifetime of the donor. The donor lifetime in radiative ET remains unchanged due to the emitted photons, while the transfer of excited energy from donor to acceptor through nonradiative ways leads to a reduced lifetime. ET rate and efficiency are two important parameters for the evaluation of an ET process. The nonradiative ET rate can also be obtained from the total decay rate of the donor in the presence of the acceptor (*k_DA_*) and the decay rate in the absence of an acceptor (*k_D_*):

(6) $$k_{\mathrm{ET}}=k_{\mathrm{DA}}\;-k_{D}$$

The ET efficiency (η_*ET*_) is defined as follows:

(7) $$\eta_{\mathrm{ET}}=\;1-\;\frac{\tau_{DA}}{\tau_{D}}=\;1-\frac{k_{D}}{k_{DA}}$$

where τ_*DA*_ and τ_*D*_ are the lifetime of the donor in the presence and absence of the acceptor, respectively. Luminescence lifetime is the reciprocal of the decay rate.

### 2.3 Photonic Effect of Chiral Nematic Structure on ET

From the above‐mentioned equations of ET rate, it is clear that the ET rate and efficiency can be methodologically regulated by changing the distance, the spectral overlap, or the relative orientations between the donor and the acceptor. For example, the ED‐ED, ED‐electric quadrupole (EQ), and EQ‐EQ ET rates scale as *R* ^−6^, *R* ^−8^, and *R* ^−10^, respectively. Maximum efficiency is achieved when donor‐acceptor dipoles are parallel‐oriented, and the efficiency is strictly zero when orientation of dipoles is perpendicular, whatever the separation between them. Besides the donor−acceptor distance and the orientation between dipoles, nanofabrication techniques have been employed to see whether ET can be regulated by means of the nanophotonic environment while leaving the donor‐acceptor pair physically and chemically unchanged.^[^ ^10^, ^26^ ^]^

As described in Equation (1), the spontaneous emission rate is determined by LDOS which represents the number of photon modes available for emission. Therefore, one may wonder whether the ET rate relates to the spontaneous emission rate, in particular, with the LDOS. If ET has a relationship with LDOS, the rate and efficiency of ET can be controlled directly by changing the photonic environment, which would be much easier to operate than altering the distance or relative orientations between dipoles. Photonic environment adjustments have already been realized in numerous studies.^[^ ^27^ ^]^ PCs are composed of periodic structures with different dielectric constants (i.e., the square of the complex refractive index) so that the photonic band gap (PBG) is formed which is an omnidirectional, propagation‐free frequency or wavelength range. An active material with a free space radiative transition, for example, a fluorophore cannot emit a photon, if it is located deeply inside a complete PBG, due to the formation of an atom‐photon bound state.

**Figure 2:**
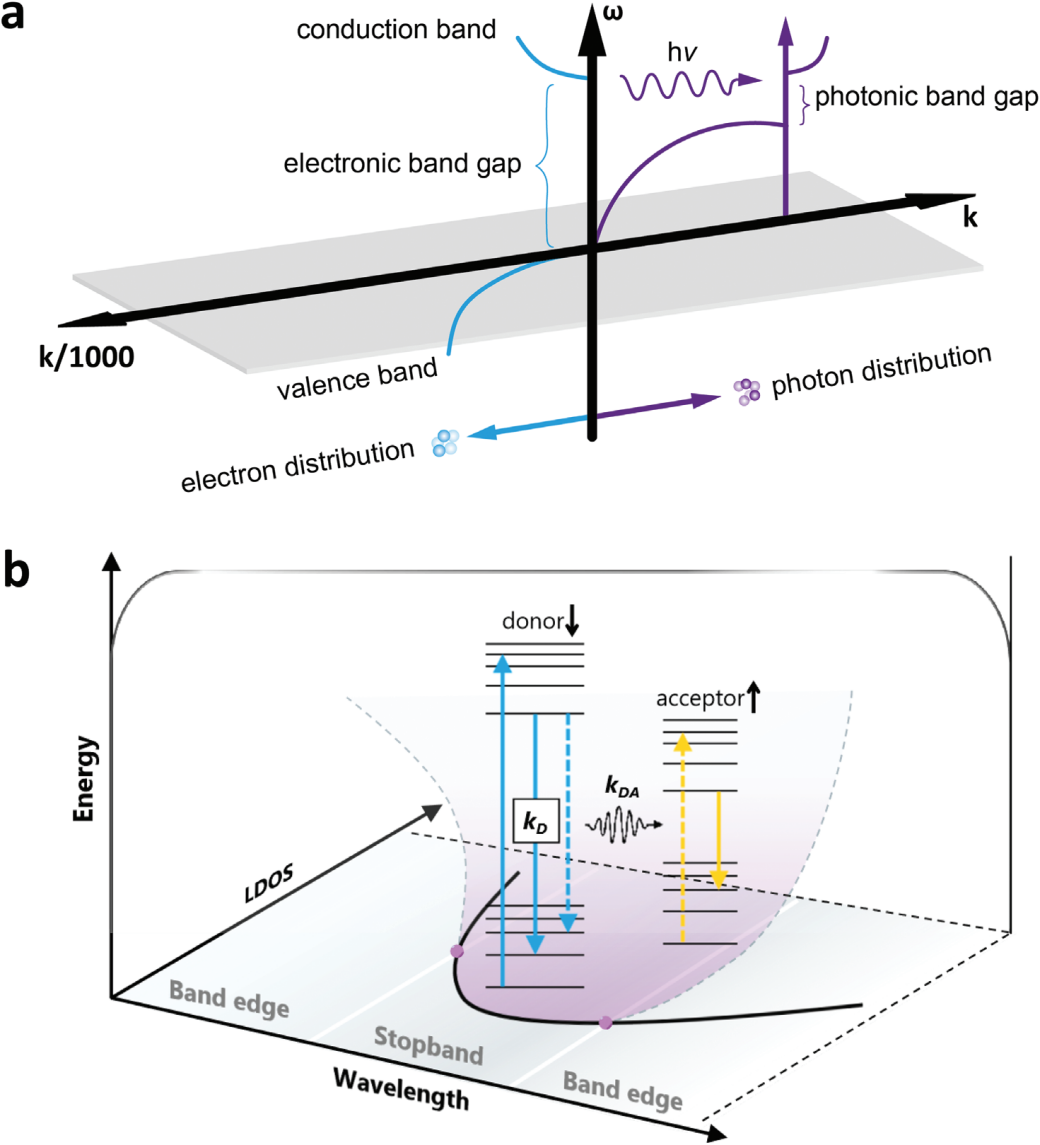
(a) The comparison of photonic band structure and electronic band structure. Right: The scheme of photon dispersion at different wave vectors (k); Left: The scheme of electron wave dispersion of a direct gap semiconductor. b) Schematic diagram showing the manipulation of luminescence by PCs. Adapted with permission.^[^ ^22a^ ^]^ Copyright 1993, Optica.

Similar to the electronic band structure formed based upon the electromagnetic scattering of electrons in the periodic crystal structure, the PBG is formed due to the Bragg scattering of photons in the periodic structures with different dielectric constants.^[^ ^22a^ ^]^ The comparison of photonic band structure and electronic band structure is shown in **Figure** **2**a. The PBG has an effect on electromagnetic wave propagation, which can be harnessed to produce distinct optical phenomena including inhibition/enhancement of spontaneous emission, omnidirectional high‐reflectivity mirrors, and low‐loss optical waveguides.^[^ ^28^ ^]^ Fluorescence is suppressed inside the PBG, while at or near the PBG edges, fluorescence can be enhanced. The reduced group velocity at PBG edges causes a longer dwelling time of the emitted photons, which can enhance stimulated emission.^[^ ^29^ ^]^

PCs can be typically classified into 1D, 2D, and 3D PCs.^[^ ^30^ ^]^ PCs have brilliant colors, due to the stopbands arising from Bragg scattering of their periodic structures. Furthermore, the stopbands of PCs can be modulated by various external stimulations including humidity, temperature, force, electric field,^[^ ^31^ ^]^ magnetic field,^[^ ^32^ ^]^ and light irradiation.^[^ ^33^ ^]^ The efficiency of ET can also benefit from the PBG effect of PCs. In detail, PCs with a stopband overlapping the donor emission band can serve as a dielectric cavity and function as a local resonance mode for donor emission propagation. The donor emission is thereby prohibited in the ET process, leaving most of the energy transferred to the acceptor (Figure 2b).^[^ ^10^, ^30^ ^]^

In this review, we focus on the 1D PCs made from N*LCs.^[^ ^31^, ^34^ ^]^ A N*LC is also called a cholesteric liquid crystal. The term cholesteric liquid crystal is used to describe the phase at first and originates from the structural characteristics of the earliest N*LCs derived from cholesterol. The word nematic denotes the state of a liquid crystal in which orientation of molecules is parallel but not aligned in a well‐defined plane. N*LCs can be the host of Ln^3+^‐doped NPs. The PBG of 1D PCs made from N*LCs can be regulated by external stimuli and further influence the spontaneous emission and ET of Ln^3+^‐doped NPs.

Specifically, the position and orientation of NPs can be controlled directly by changes in external stimuli. For Ln^3+^, although the spectral features remain largely unchanged, the ET efficiency can be affected by the composition structure of the Ln^3+^‐hosting medium.^[^ ^35^ ^]^ This can lead to the polarized emission of individual NPs due to the intra‐ion transition features and the local site symmetry of Ln^3+^.^[^ ^36^ ^]^ However, polarization‐dependent properties usually lack tunability and are not often observed, given the random orientations of these anisotropic NPs. The 1D PCs made from N*LCs are thereby promising hosts that can combine the unique optical, electronic, and magnetic properties of Ln^3+^‐doped NPs with a long‐range ordered structure and facile responses toward external stimuli of assemblies.^[^ ^37^ ^]^ Some examples concerning the external control methods will be given in Section 4, including electric field, light, additives, ultrasonics, and humidity. The PBG of the chiral nematic structure in 1D PCs made from N*LCs can be regulated under external stimuli, leading to a change in spontaneous emission and ET.

## 3 Formation and Responsive Regulation of Chiral Nematic Structure

### 3.1 Formation, Optical Functions, and Characteristics of Chiral Nematic Structure

Among the wide research interests on chirality, chiral nanoarchitectonics is an emerging concept and touches upon several aspects including the manipulation of an atom/molecule, the reaction process, and the product structure. In this review, we focus on the chiral nanoarchitectonics in liquid crystals and their roles in manipulating ET with Ln^3+^. The chirality of N*LCs has some extent of flexibility due to the partially fluidic nature of the LC materials. As mentioned above, there are two kinds of N*LCs, depending on their fabrication processes. The small‐molecule N*LCs are composed of chiral molecules that form a helical structure. The N*LCs of CNCs with the helical structure are formed by the self‐organization of CNCs into a chiral nematic liquid crystalline phase (Figure 1b).

Small‐molecule N*LCs usually consist of rod‐like units and chiral dopants. The units will orient in one direction in each plane. In the perpendicular direction to the plane, the units will arrange in a chiral helical structure upon doping with chiral dopants. The helical pitch is defined as the distance over which a full rotation of 360° is finished for the unit orientation. Small‐molecule N*LCs exhibit a selective structural color, depending on the periodicity and handedness of the helices.^[^ ^38^ ^]^ Given the periodic refractive index changes caused by continuous rotation of the director of LC units, N*LCs demonstrate great potential in polarizers, optical filters, and chiral photo displays. By controlling the ratio of chiral dopants or applying an electric field, the helical pitch of N*LC can be flexibly regulated as shown in Figure 1c.^[^ ^14^, ^39^ ^]^

Helices are also abundant in biomaterials, for example, the CNCs produced by hydrolyzing native cellulose generally present a rod‐like morphology and a spirally stacked structure that can assemble into a N*LC phase under a critical concentration. The spirally twisted structure is one indispensable factor for the formation of the chiral nematic structure.^[^ ^40^ ^]^ Revol et al. first studied the helicoidal self‐ordering structure in both suspensions and films of cellulose in 1992.^[^ ^41^ ^]^ CNC films with chiral nematic structures will reflect visible light and present vivid iridescent structural colors if the pitch is in the visible region.^[^ ^42^ ^]^ The wavelength of reflected light (λ) by chiral nematic structures can be expressed as in Equation (8), which depends on the refractive index *n*, the pitch *P*, and the reflection angle θ with respect to the film surface.^[^ ^43^ ^]^ The PBG can be obtained from the reflected spectra and tuned by changing the pitch according to the equation:

(8) λ=nPsinθ

It is indispensable to control the self‐assembly process of N*LCs for the design of advanced materials. Self‐assembly of CNCs into chiral nematic CNC films has been described as a three‐stage process by Dumanli et al., which includes i) initial isotropic suspension, ii) a viscous gel phase formation, and iii) film formation.^[^ ^42^, ^44^ ^]^ A diagram depicting the phase behavior of CNCs at each stage is illustrated in Figure 1b, where plots in the middle figure describe relationships between CNC concentrations with phases (blue line) and with pitches (yellow line).^[^ ^19^ ^]^

Besides the aforementioned unique optical properties originating from the periodic helical structure, e.g., optical rotation and circular dichroism, optical functions including hyper‐reflection^[^ ^45^ ^]^ and chirality inversion^[^ ^46^ ^]^ can also be seen in chiral nematic structures. Moreover, light can be slowed down and even trapped in the close vicinity of PBG in a chiral nematic structure. Thus, micro and nano lasers can be designed by doping gain into N*LCs media, such as an organic dye, or semiconductor.^[^ ^47^ ^]^ Chiral nematic CNC suspensions can be used as the soft template to direct the chiral assemblies of other nanoparticles and endow the hybrid system with a chiral feature. Due to the chiral nematic structure, CNC films reflect left‐handed CPL and present a positive circular dichroism signal. Transparent CNC films enable controlled optical diffusion, UVB (280–320 nm) shielding, and radiative cooling.^[^ ^48^ ^]^ The CNC films with PBG matched to the emission can function as an efficient resonator for low‐threshold multimode lasing.^[^ ^49^ ^]^

Many characterization methods have been used to describe the formation of chiral nematic structures. Compared with the usual trichromatic red‐green‐blue (RGB) imaging, the optical response is divided into many more bands in hyperspectral imaging, which can provide more elaborate information. Thus, hyperspectral imaging, coupled with optical microscopy, has been used to investigate the optical response of defects in CNC films.^[^ ^50^ ^]^ Small‐angle neutron scattering is a powerful technique, particularly in studying colloidal assemblies with distinct materials. Van Rie et al.^[^ ^51^ ^]^ presented a detailed small‐angle neutron scattering study on the self‐assembly behavior of mixed CNC‐gold nanorod suspensions. Frka‐Petesic et al.^[^ ^52^ ^]^ retrieved the co‐assembly pathway of composite CNC films from their angular optical response using angle‐resolved optical spectroscopy.

### 3.2 Responsive Regulation of Chiral Nematic Structure

**Figure 3:**
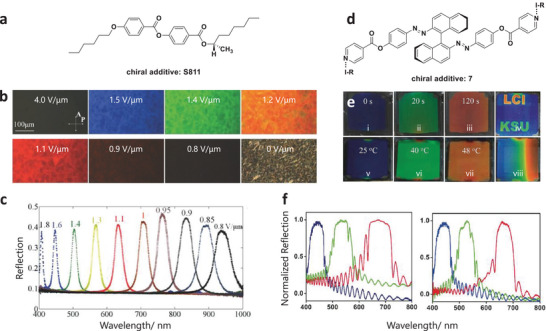
Representative examples of chiral nematic structure controlled by electric field and light irradiation. a) Molecular structure of the left‐handed chiral additive S811. b) Electric field‐induced texture changes of the cholesteric mixture (CB7CB:CB6OCB:5CB:S811 = 30:20:46:4, in weight units) from an unwound nematic state to helicoidal states with reflected RGB colors, to two infrared‐reflective states, and to a fingerprint state. c) Reflection spectra of a cholesteric cell under different amplitudes of the electric field. Reproduced with permission.^[^ ^53a^ ^]^ Copyright 2015, Wiley‐VCH. d) Molecular structure of the chiral additive 7. e) Light (i–iv) and temperature (v–viii) induced image changes of a thick planar cell filled with switch 7 in E7. RGB colored image by light excitation (e, iv) and constructing temperature gradient (e, viii). f) Light (left) and temperature (right) induced reflection spectra. Reproduced with permission.^[^ ^56^ ^]^ Copyright 2018, Wiley‐VCH.

Compared to other passive methods, the fascinating responsive methods to control the structure of N*LCs are by electric and magnetic fields. Xiang et al. ^[^ ^53^ ^]^ reported a cholesteric material with a unique helicoidal structure, for which its reflection can be regulated by an electric field. The designed cholesteric material was formulated by mixing two dimeric LCs CB7CB (1′,7′‐bis(4‐cyanobiphenyl‐4′‐yl)heptane) and 4CB6OCB (1‐(4‐cyanobiphenyl‐4′‐yl)−6‐(4‐cyanobiphenyl‐4′‐yloxy)hexane) with a standard LC‐5CB (4‐cyano‐4′pentylbiphenyl). Followed by doping with S811 (**Figure** **3**a), the mixture was then filled into a sandwiched planar cell. The uniform nematic structure can be developed under a strong electric field. Then the structure can undergo a sequence change with the decrease of the field from 4 V µm^−1^ to 0 V µm^−1^. The polarizing microscopy (POM) images (Figure 3b) and the reflection spectra (Figure 3c) both verified the tunability. The use of a magnetic field also was reported by Wang et al. ^[^ ^54^ ^]^ to control the position and orientation of LCs structure due to the different magnetic susceptibility between tactoids and isotropic phases after doping with superparamagnetic NPs.

Light or heat control of N*LCs structure was also explored recently.^[^ ^34^, ^55^ ^]^ Wang et al. ^[^ ^56^ ^]^ designed a halogen‐bonded light‐driven chiral molecular switch 7 (the structure is shown in Figure 3d) and doped it into the achiral nematic liquid crystal E7. The resulting N*LCs were then filled into a planar cell. The cell exhibited different reflection colors due to the change of pitch by controlling the light irradiation time or heat (Figure 3e). Light irradiation causes photoisomerization of the chiral molecular switch 7, resulting in a change in the helical twisting power. Furthermore, heat‐driven RGB reflections from blue to red were also observed (Figure 3f). By controlling the ratio of chiral dopants, the PBG of N*LCs also could be flexibly regulated.^[^ ^57^ ^]^ More detailed information about the LCs additives, controlling methods, and the related range of reflection or pitch are summarized in **Table** **1**.

**Table 1:** Summary of the composition, tuning methods, reflecting range or pitch of some common N*LCs

LCs	Chiral additives	Controlling methods	Range of reflection [*R*] or pitch [*P*]	Reference
SLC1717	chiral dopant‐isosorbide derivative	ratio	R 2000 nm	[67]
5CB	cholesterol‐carrying gold nanorods	ratio	P 2.4–3.4 𝜇m	[68]
5CB	chiral colloidal particles	distortions		[69]
	DNA origami filaments	molecular designs	P 10–80 𝜇m	[70]
E7	a chiral binaphthyl molecule and light‐driven molecular motors	light		[71]
SLC1717	light‐driven chiral fluorescence capabilities	light	R visible to infrared	[72]
E7	photoresponsive tristable chiral molecule	light	R visible to infrared	[73]
5CB/PCH5	photochromic hydrazone	light	P 1900–5000 nm	[74]
7CB/ 5CB	heliconical cholesterics	electrical field	R UV to visible and infrared	[53a]
E7/S811	a chiral azobenzene photoswitch/UCNPs	light	R blue to red	[75]
E7	photoactive halogen‐bonded chiral switch	light	R blue to red	[56]
E7/S811/R811	photoresponsive tristable chiral switch	light	R visible to infrared	[73]
6OBA	benzoic acid metal binding sites	calcium	R green to blue	[76]

Self‐assembly of CNCs is of fundamental importance to form chiral nematic structures and to perform fascinating properties, which can be controlled by many internal influences, such as the surface property of CNCs,^[^ ^58^ ^]^ ionic strength of the suspension,^[^ ^59^ ^]^ effects of water evaporation, initial CNC concentration and the casting surface of CNC suspension. The self‐assembling process can also be affected by external factors including additives, ultrasonic treatment, and other auxiliary methods.^[^ ^60^ ^]^ For example, Chu et al. ^[^ ^61^ ^]^ employed the freeze‐casting method to get anisotropic aerogels with long‐range ordering structures. The CNCs orientation can be formed due to the shear flow field of ice crystals and fixed by sublimation of the ice template. Assembly tuning by additives has been seen in glycerol,^[^ ^62^ ^]^ sodium chloride, ionic liquid, poly(ethylene glycol) diacrylate,^[^ ^63^ ^]^ poly(N‐isopropyl acrylamide),^[^ ^64^ ^]^ poly(vinyl alcohol) (PVA),^[^ ^65^ ^]^ polysaccharides^[^ ^66^ ^]^ etc. (summarized in **Table** **2**). Notably, the pitch is changed upon changing the amount of additives, which is a passive modulation.^[^ ^42^ ^]^

**Table 2:** Summary of additives explored in controlling the self‐assembly of N*LCs of CNCs

Additives	Controlling methods	Range of reflection [*R*] or pitch [*P*]	Reference
glycerol	ratio/humidity /mechanical compression	P 225–400 nm	[62a]
glycerol	Ratio	R 450 to 850 nm	[62b]
glycerol	Ratio/humidity	R 425 to 650 nm	[62c]
poly(ethylene glycol) diacrylate	Ratio	P 2.5‐3.1 𝜇m	[63]
dimethy‐lmyristylammonio propanesulfonate (DMAPS)	Ratio	R 525 to 825 nm	[77]
poly(ethylene glycol)	Ratio	R 320 to 665 nm	[78]
poly(*N*‐isopropyl acrylamide)	ratio/heating/humidity	R 350 to 550 nm	[64]
poly(vinyl alcohol)	ratio	R 450 to 625 nm	[65a]
poly(vinyl alcohol)	ratio	R 560 to 726 nm	[65b]
poly(vinyl alcohol)/glutaraldehyde	ratio	R 350 to 800 nm	[79]
polysaccharides	ratio	R 300 to 800 nm	[66]
ureidopyrimidinone (UPy)	ratio	R 525 to 1050 nm	[80]
1,2‐bis(trimethoxysilyl) ethane	ratio	R 450 to 850 nm	[52]
PMTAC	anion exchange	R 500 to 700 nm	[81]
elastomers (ethylacrylate and 2‐hydroxyethyl acrylate)/	stretch	R 475 to 675 nm	[82]
poly(ethyl acrylate) elastomer	stretch	R 450 to 650 nm	[83]
acrylamide (AAm)	solvent/pH/temperature	R 525 to 1200 nm	[84]
H_2_O	concentration	R 475 to 525 nm	[85]
urea–formaldehyde resin	press	R 500 to 630 nm	[86]
polyacrylamide (PAAM)	press	R 178 nm	[87]
PDMS polymer	strain	P 390–500 nm	[88]
4‐cyano‐4‐pentylbiphenyl (5CB)	temperature/electric field		[89]
photoactive polymer/PEG	light/humidity	P 328–422 nm R 514–805 nm	[90]
polyacrylamide (PAAm) NaCl	magnetic field		[14, 91]
Fe_3_O_4_ NPs	magnetic field	P 206–302 nm	[92]
beycostat NA surfactant	electric field	P 2–9 𝜇m	[93]

**Figure 4:**
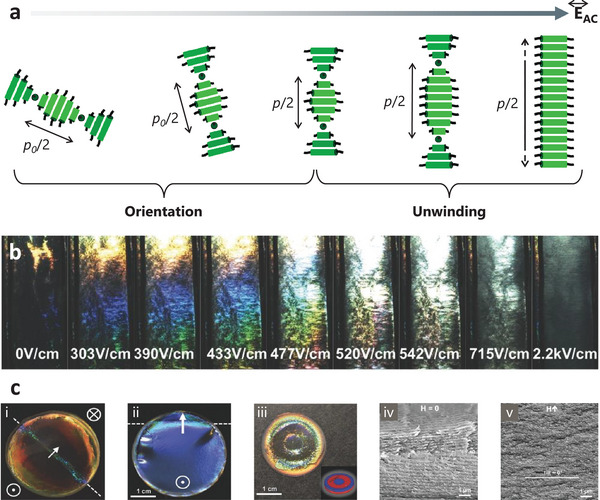
Representative examples of self‐assembly of CNCs controlled by a,b) electric, and c) magnetic fields. a) A schematic diagram showing the sequential orientation and unwinding of cholesteric structure upon electric field increase. b) Iridescence of a cholesteric CNC suspension upon the increase of electric field. Reproduced with permission.^[^ ^93^ ^]^ Copyright 2017, Wiley‐VCH. c) Representative macroscopic images of the CNC film that was: i) and ii), on different sizes of magnets (the later the larger magnets); iii), on a polydomain magnet; SEM: iv, in the absence of a magnetic field (H = 0); v, in the presence of a magnetic field, denoted by H↑. Reproduced with permission.^[^ ^91^ ^]^ Copyright 2017, John Wiley and Sons, Inc.

Compared to the passive modulating methods described above, stimuli‐responsive self‐assembly of CNCs is more attractive due to their continuity and reversibility. Temperature, light, electric field, and magnetic field are representatives of these stimuli to assemble CNCs (Figure 1c). Due to the diamagnetic anisotropy of the individual C─C, C─O, C─H, and O─H bonds from CNCs and the presence of both permanent and induced dielectric anisotropy, magnetic^[^ ^94^ ^]^ and electric fields^[^ ^95^ ^]^ can be applied to align CNC rods in suspension. Electric‐field control can be seen in recent work by Frka‐Petesic et al.,^[^ ^80^ ^]^ where the use was shown to reorient the chiral nematic phase and tune the pitch at intermediate fields. The application of an electric field leads to the unwinding of the chiral structure and results in purely aligned CNCs. The degree of alignment CNCs can be regulated by the strength and frequency of the applied electric field: which can be 88% at 800 V mm^−1^ and 2000 Hz. The methods of iridescence observation and laser diffraction can be employed to monitor the alignment process qualitatively and quantitatively, respectively (**Figure** **4**a,b).^[^ ^93^ ^]^

Magnetic‐field control has also been successfully exploited.^[^ ^14^, ^96^ ^]^ Taking a work by the Frka‐Petesic group as an example, magnetic fields were found to control the self‐assembly of CNCs into colorful films, depending on the direction and angle of the applied field.^[^ ^91^ ^]^ Figure 4c shows the macroscopic and SEM images of a CNC film formed under a small commercial magnet (≈ 0.5–1.2 T). Magnets of different sizes were arranged under the CNC suspension (Figure 4c, i,ii). The magnet with a patterned polydomain can be applied to prepare the CNC film with a radial pattern (Figure 4c, iii). The chiral structure of the film was along with various tilts and angles in the absence of the magnetic field (Figure 4c, iv). Highly ordered structures were formed when applying the magnetic field (Figure 4c,v). This study demonstrates the effectiveness of a magnetic field to improve the orientation and the homogeneity of chiral structure.^[^ ^42^, ^91^ ^]^

**Figure 5:**
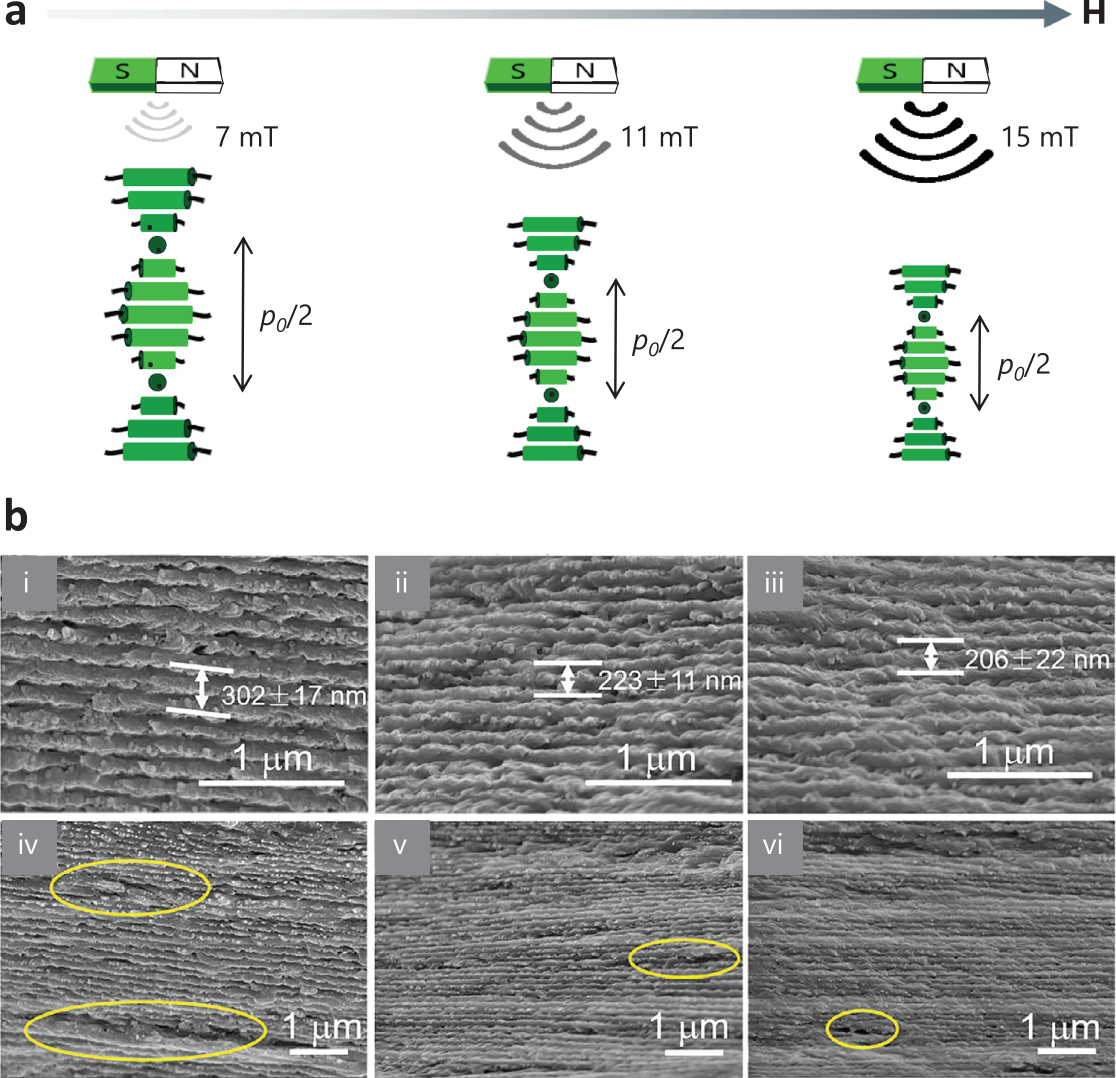
Controlling the Fe_3_O_4_/CNCs self‐assembly by an ultrasmall magnetic field. a) A schematic diagram showing the tuning of the self‐assembly process and the pitch of Fe_3_O_4_/CNCs by ultrasmall magnetic fields. b) SEM images of Fe_3_O_4_/CNC films under 7, 11, and 15 mT magnetic fields: i–iii) higher magnification; iv–vi) lower magnification. The reduced defects (indicated in yellow circles) in the image suggest improved structural uniformity is presented with the increase of the applied magnetic field. Reproduced with permission.^[^ ^92^ ^]^ Copyright 2020, American Chemical Society.

Notably, one strategy to further improve the responsiveness of N*LCs of CNCs to magnets is to decorate the CNCs with superparamagnetic Fe_3_O_4_ NPs (termed Fe_3_O_4_/CNCs). Dhar et al.^[^ ^97^ ^]^ employed a low magnetic field (≈ 50–60 mT) to align the CNCs decorated with high content of Fe_3_O_4_ (51 wt.%). Along with this strategy, a subsequent dispersion of Fe_3_O_4_/CNCs was introduced on the CNCs, which was emphasized as a key step to improve the sensitivity toward magnetic fields. As shown in **Figure** **5**, the pitch can be tuned from 302 nm to 206 nm by controlling the ultrasmall magnetic field from 7 mT to 15 mT.^[^ ^92^ ^]^ This ultrasensitive magnetic particle tuning method has been employed by Hausmann et al.^[^ ^98^ ^]^ to fabricate functional microparticles, where the controlled optical modulation and sensing were achieved by the contactless, remote magnetic stimuli.

Pressure, tension, and temperature have also been explored for the control of CNC‐based N*LCs. The MacLachlan lab and the Zhao group reported hydrogels fabricated either from CNCs or hydroxypropyl cellulose, which can respond to multiple external stimuli including solvent, pH, pressure, tension, and temperature. Notably, the hydrogel developed by Zhang et al.^[^ ^99^ ^]^ can output the stimuli as electric resistance signals due to the incorporation of carbon nanotube additives. It can also perform optical sensing of the stimuli through color migration caused by internal structural changes. Thus, this hydrogel showed great application prospects as a multifunctional electrical skin.^[^ ^84^, ^99^, ^100^ ^]^ Another highly stretchable, transparent, and ionic conductive CNCs/polyvinyl alcohol hydrogel was fabricated by Wang et al., ^[^ ^101^ ^]^ which exhibited a fast and stable response to temperature, compressive pressure, and tensile strain. The MacLachlan lab also reported the successful incorporation of CNC‐based N*LCs into an elastomer composite.^[^ ^82^ ^]^ The composite was not only elastomeric but also chromatic when applying mechanical stress.^[^ ^102^ ^]^ These elastomers could find applications such as security features, pressure, and fracture sensors.

Many factors can be utilized to control the self‐assembly process of CNCs, and such controls usually benefit properties of CNC films including electrical, thermal, and mechanical performance,^[^ ^97b^ ^]^ as can be seen in the following examples. Alignment of Ag‐coated fibrillated celluloses in a polymer matrix can not only increase dielectric permittivity but also enhances anisotropic optical properties.^[^ ^103^ ^]^ Tatsumi et al.^[^ ^96a^ ^]^ designed anisotropic composites with improved thermodynamic properties by applying a rotating magnetic field to control the orientation of CNCs in hydroxyethyl methacrylate. The orientation can be further locked by photo‐induced polymerization.^[^ ^96^, ^104^ ^]^ High‐strength and anisotropy adhesives based on CNCs were developed by Tardy et al.^[^ ^105^ ^]^ upon confined evaporation‐induced self‐assembly. The inherent strong CNCs aligned into rigid, nematic order with significant anisotropy of adhesion, so that new emergent properties are therefore expected.

The chiral nematic structure of CNCs can also be the template to facilitate the synthesis of materials with specific shapes or unique properties such as the synthesis of chiral carbon dots.^[^ ^106^ ^]^ Cellulose can also serve as a nanoreactor to synthesize 1D nanocrystals covering Au, Pt, BaTiO_3_, CdSe, TiO_2_, Fe_3_O_4_, NaYF_4_:Yb, Er.^[^ ^107^ ^]^ The optical structures can also be imprinted onto the chiral biomaterials, which boosts a wide range of photonic applications, such as stereoscopic displays, chiral polarizers, polarization encoding, colorimetric chiral biosensing, etc.^[^ ^108^ ^]^

## 4 Lanthanide‐Doped Nanoparticles in Chiral Nematic Structure

### 4.1 Preparation of the Hybrid Systems

The hybrid system combination of light‐emitting materials and chiral nematic structure exhibits distinctive optical features. Quantum dots,^[^ ^109^ ^]^ gold nanoparticles,^[^ ^110^ ^]^ dyes,^[^ ^111^ ^]^ and carbon dots^[^ ^65^, ^112^ ^]^ are representative light‐emitting materials that can assemble with CNCs. For example, Guo et al. demonstrated a conceptual approach toward environmentally sustainable stimuli‐switchable lasers composed of CNCs and dye‐doped water‐soluble polymers.^[^ ^49^ ^]^ Hou et al.^[^ ^113^ ^]^ described a new water and formaldehyde‐sensitive membrane with a chiral structure by combining fluorescent molecules with CNCs. Despite the efforts devoted to the development of photoluminescent CNC‐based materials, few examples can be seen with the fabrication of CNC hybrids with lanthanide‐activated NPs, for example, upconverting NPs (UCNPs), which absorb two or more photons with low energy and emit higher energy photons.^[^ ^114^ ^]^ UCNPs, particularly Yb^3+^, Er^3+^‐doped NaYF_4_, where the 980 nm exciting photon is absorbed by the sensitizer Yb^3+^ and then transferred to the ^4^I_11/2_ state of Er^3+^,^[^ ^115^ ^]^ is one of the most promising upconverting luminescent nanomaterials due to the low photon energy and high upconversion efficiency.^[^ ^116^ ^]^ Nguyen et al. reported the first chiral photonic films prepared by co‐assembling CNCs with NaYF_4_:Yb^3+^, Er^3+^ nanorods, which exhibited upconversion emission and tunable photonic chiral activity simultaneously.^[^ ^117^ ^]^

For the small‐molecule N*LCs, a certain amount of modified lanthanide‐containing NPs is usually doped directly into the cholesteric LC system. The incorporated Ln^3+^‐based NPs are controlled to cause weak (or not any) perturbation on the LC matrix. For example, methoxy poly(ethylene glycol) silane can be used to modify the oleic acid‐capped UCNPs, which will relieve the distortion of the LC director around the UCNPs.^[^ ^37^, ^118^ ^]^ Besides modifications on the particle surface, the control of other parameters such as the final particle volume fraction or the ionic concentration have also been exploited to obtain a stable liquid crystalline phase.^[^ ^119^ ^]^

CNC hybrids with lanthanide‐doped NPs were usually obtained by the above‐mentioned evaporation‐induced self‐assembly method from the homogeneous CNC/NPs dispersion.^[^ ^62^, ^120^ ^]^ Various types of interactions such as van der Waals force, hydrogen bonding, electrostatic and steric interactions, molecular dipole effect, solvophobic interaction, depletion, and capillary action, are involved in the self‐assembly process which can modulate the structural organization of the matrix.^[^ ^121^ ^]^ Specifically for CNCs self‐assembly, hydrogen bonding, and electrostatic forces are the dominant forces.^[^ ^122^ ^]^ As discussed, CNCs with crystalline domains are commonly made by hydrolyzing the amorphous regions of cellulose with sulfuric acid. Thus, the CNCs will be hydrophilic and the colloid stable due to the negatively charged sulfate half‐ester group (zeta potential, −40.7 mV) on the surface, leading to the occurrence of self‐assembly.^[^ ^42^ ^]^ Modifications of NPs are also needed for compatibility with CNCs. For example, the chiral structure of CNCs can be disrupted in the mixture of the hydrophobic oleic acid‐capped UCNPs and hydrophilic CNCs. Therefore, an amphiphilic stabilizer, e.g., polyvinylalcohol has been used to modify the hydrophobic UCNPs.^[^ ^117^ ^]^ As a result, a uniform CNC film exhibiting iridescent colors and upconverted photoluminescence can be obtained.^[^ ^62c^ ^]^

### 4.2 Responsive Regulation of ET of Lanthanide‐Doped Nanoparticles in Chiral Nematic Structure

As discussed in Section 2, spontaneous emission and ET can be affected by the LDOS induced by the PBG effect of PCs.^[^ ^123^ ^]^ In detail, the rate of spontaneous emission can be tuned by the LDOS as described in Equation (1). Through such control, LDOS can demonstrate an indirect effect on the ET process. Theoretically, ET efficiency could be zero through the extreme increase of the spontaneous emission rate; likewise, ET efficiency can be maximized by reducing the LDOS and the consequent spontaneous emission rate.^[^ ^26^ ^]^ We focus on the PBG effect on spontaneous emission and ET of lanthanide‐doped NPs dispersed in N*LCs. In addition, CPL will originate in the selective reflection from the helical structure of the N*LCs.^[^ ^124^ ^]^ Like the chiral nematic structure, hybrid materials composed of lanthanide‐doped NPs and chiral nematic structure can be responsively controlled by stimuli like humidity, electric field, magnetic field, temperature, and light. Thus, the photonic effect of 1D PCs with chiral nematic structure on spontaneous emission and ET is now summarized under different regulation methods.

#### 4.2.1 Electric Field Regulation of ET

Polarized luminescence from anisotropic emitters, such as quantum rods,^[^ ^125^ ^]^ semiconductor nanowires,^[^ ^126^ ^]^ and organic dyes,^[^ ^127^ ^]^ has been well‐studied. However, polarized photoluminescence from Ln^3+^ ions shows a distinguishing nature. Ln^3+^ ions are featured with abundant energy levels, multiple transitions, and sublevel degenerations in both upconversion and downshifting systems.^[^ ^37^, ^128^ ^]^ The emission will be polarized in the crystalline host matrices with specific site symmetry, leading to the orientation‐dependent line profiles. In addition, Ln^3+^ emission spectra are relatively independent of the size and morphology of the nanoemitters, which makes Ln^3+^ ions the ideal model to carry out the orientation analysis compared to other anisotropic emitters.^[^ ^39^, ^119^, ^129^ ^]^ A representative example can be seen in NaYF_4_:Eu nanorods which have been used as a model system to measure the 3D orientation from polarized luminescence with high accuracy.^[^ ^130^ ^]^

A prerequisite for acquiring polarized luminescence is to achieve a uniform orientation of a single crystal or small crystallites doped with Ln^3+^. Electrical‐responsive LCs recently emerged as host media to regulate the orientation of the nanorods in LC cells. As a result, the orientation, crystalline matrices, as well as optical emission, could be regulated by the applied electric field. Mundoor et al.^[^ ^37^ ^]^ designed a dispersion system of rod‐like UCNPs based on nematic LC. As the LC host media was switched by the electric field, the UCNPs would orient along the LC director and barely perturb the structure of the LC matrix. The ET efficiency from Yb^3+^ to Tm^3+^ and Er^3+^ depended on the local crystal symmetry which induces polarization anisotropy of UCNP emission because of the fine structure splitting of the energy levels.^[^ ^37^, ^131^ ^]^ Kim et al.^[^ ^119^ ^]^ used electrically regulated LC to control the orientation of LaPO_4_:Eu^3+^ nanorods and studied the polarized luminescence. There are different emission profiles depending on the orientation of nanorods. Thus, the system can be used to measure the local shear rate in a flowing liquid based on the fact that the orientation of flowing nanorods tends to align with the shear strain.

**Figure 6:**
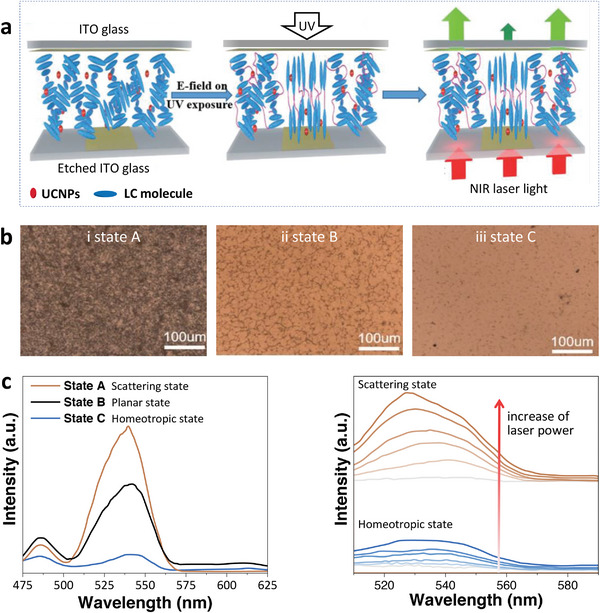
a) Schematic diagram of UC luminescent patterning fabrication. b) POM images of UCNPs/LC films in i) state A: scattering state, ii) state B: planar state, and iii) state C: homeotropic state; c) Left: The emission spectra of UCNPs/LC films in three states; Right: The variation tendency of emission in scattering state and homeotropic state with the increasing intensity of 980 nm laser power. Reproduced with permission.^[^ ^118^ ^]^ Copyright 2017, Wiley‐VCH.

Ye et al.^[^ ^118^ ^]^ also evaluated the luminescence of UCNPs in the LC network host with and without the electric field. The fabrication of the UC luminescence micropatterns is shown in **Figure** **6**a. When the LC was in the scattering state with focal conic texture where the helical axes aligned along the substrates (Figure 6b, **s**tate A), the 980 nm excitation light is scattered and reflected by the LC, leading to a longer zigzag travel path. Thus, UCNPs dispersed in the LC are more excited by 980 nm radiation, which results in enhanced emission. As for the planar state (Figure 6b, state B) in which helical axes were aligned perpendicular to the substrate, part of the 980 nm excitation light passes through the host without scattering, leading to slightly weaker emission compared with the emission of the scattering state. The LC is in the homeotropic state (Figure 6b, state C) when an electric field was applied, making LC molecules align perpendicularly to the substrates. The 980 nm excitation light passes through the LC host without scattering, giving rise to decreased light absorbed by UCNPs, and thus leading to a largely weakened UC emission. The emission spectra of the UCNPs in LC films at these three states are summarized in Figure 6c, left. Furthermore, the laser power dependence of intensity for different states is verified in Figure 6c, right. The intensity in state A increases rapidly with the increase of laser power, while in state C where the excitation light can be barely absorbed, the intensity increases slightly with the increase of laser power.^[^ ^118^ ^]^ In addition, Tong et al.^[^ ^132^ ^]^ realized the reversible regulation of the UC emission intensity by only controlling the applied voltage. They loaded UCNPs in an LC matrix, in which the LC was originally in the homeotropic state. The excitation light was scattered in the LC matrix with focal conic texture after applying the electric field, leading to enhanced emission. Under the electric field of 4.6 V 𝜇m^−1^, the enhancement factor was 6–8. The way to tune the luminescence by changing light scattering power is facile, compared to the demanding synthesis and modification of UCNPs.

**Figure 7:**
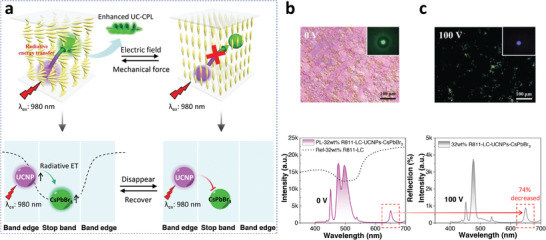
a) Upconverted circularly polarized luminescence (UC‐CPL) of N*LCs incorporated with UCNPs and CsPbBr_3_, through the radiative ET process. b) Top: POM image showing the planar texture of N*LC. Bottom: Upconverted emission exhibited by both CsPbBr_3_ and UCNPs. c) The radiative ET process from UCNPs to CsPbBr_3_ was switched off by the applied voltage of 100 V. Top: POM of N*LC under the voltage of 100 V. Bottom: The emission at 646 nm of UCNPs at 100 V had decreased by 74%. The inset pictures at the top of Figures (b) and (c) show the emission of N*LC. Reproduced with permission.^[^ ^57^ ^]^ Copyright 2020, Wiley‐VCH.

Electric field‐regulated ET in small‐molecule N*LCs has also been realized. Yang et al.^[^ ^57^ ^]^ incorporated NaYF_4_:Yb/Tm UCNPs and CsPbBr_3_ perovskite nanocrystals into N*LCs constructed from SLC1717 and R(S)811. In the system, UCNPs and CsPbBr_3_ served as the donor and acceptor, respectively. The PBG is ≈500 nm when the weight ratio of chiral additives R811/SLC1717 was set as 32%. Thus, the emission (450 nm, 475 nm from UCNPs; 495 nm from CsPbBr_3_) was located at the edge and center of the PBG, respectively (**Figure** **7**a). The enhanced UCNPs emission benefiting from the band edge effect could further increase the emission of CsPbBr_3_ through the ET process (Figure 7b). The transfer from UCNPs to CsPbBr_3_ was by radiative rather than nonradiative ET because the donor (UCNPs) lifetimes at 450 and 475 nm (quenched by the acceptor CsPbBr_3_) were essentially unchanged. In addition, the radiative ET process can be turned off by applying the electric field, which changes the orientation of the N*LCs and suppresses the emission of UCNPs (Figure 7c). Moreover, the radiative ET process could be recovered after applying mechanical stress. This work realized stimuli‐responsive ET between UCNPs and CsPbBr_3_ in small‐molecule N*LCs.

#### 4.2.2 Light Regulation of ET

**Figure 8:**
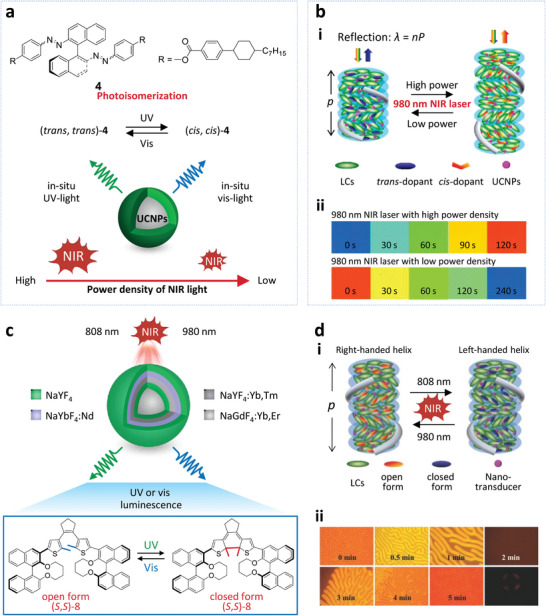
a) Top: Chemical structure of the light‐driven chiral molecular switch 4. Bottom: Schematic diagram showing photoisomerization of the molecular switch 4. The in situ UV and visible light from UCNPs (β‐NaGdF_4_: 70 mol% Yb^3+^, 1 mol% Tm^3+^@β‐NaGdF_4_) excited by 980 nm laser triggers the photoisomerization of the chiral switch 4, leading to the selectively reflective wavelength. b) i: Schematic mechanism of the selective reflection for the helical structure. ii: The polarized reflective mode microscope images of the LCs in a thick planar cell excited by a 980 nm laser with high (2 W mm^−2^) and low (0.15 W mm^−2^) power density. Reproduced with permission.^[^ ^75^ ^]^ Copyright 2014, American Chemical Society. c) Schematic diagram showing the core‐shell structure of the UCNPs (β‐NaGdF_4_:Yb, Er@β‐NaYF_4_@β‐NaYF_4_: Yb, Tm@β‐NaYbF_4_: Nd@β‐NaYF_4_) and the chemical structure of the molecular switch (S, S)−8. d) i: Schematic mechanism of the reversible handedness inversion for the helical structure. ii: The microscopy pictures of the LCs mixture in a homeotropic cell showing the handedness inversion from right‐handed (0‐1 min) to left‐handed (3–5 min). Pictures in the last column represent the transient nematic phase and its corresponding conoscopic observation (2 min). Reproduced with permission.^[^ ^46^ ^]^ Copyright 2015, Wiley‐VCH.

ET of lanthanide‐doped NPs in cholesteric liquid crystals can also be regulated by changing the power density or wavelength of the excitation light. Wang et al. ^[^ ^75^, ^133^ ^]^ fabricated a system by doping UCNPs and a light‐driven chiral molecular switch into two commercial LCs, the LC E7 and S811. The molecular switch **4** can transform between its trans and cis isomers upon excitation by UV or visible light (**Figure** **8**a). According to the relationship between the intensity and excitation power density for UCNPs, the visible emission at 475 and 450 nm correspond to the three‐ and four‐photon ET processes, respectively, while the ultraviolet emissions at 365, 343, and 290 nm are due to the four‐ and five‐photon ET processes. Thus, the UV emission can increase greatly, under excitation by a 980 nm laser with high power density, whereas the visible emission dominates at low power density. Note that the UV emissions have spectral overlap with the π→π* absorption bands of the trans‐chiral switch, and the visible emissions overlap with the *n*→π* absorption bands of the cis‐chiral switch (Figure 8a), making it possible for the effective ET from UCNP donor to chiral switch acceptor and hence gain control on the reflected light. Subsequently, reversible regulation of reflections from blue to red wavelengths has been realized in the helical superstructure only by decreasing the power density of the 980 nm near‐infrared laser (Figure 8b).

Wang et al.^[^ ^46^ ^]^ further prepared a mixture by doping the thermally driven chiral molecular switch (S, S)−8 and UCNPs into the commercial achiral LC host E7. The UCNPs doped with Tm^3+^ and Er^3+^ can act as nano transducers triggering the transformation of the chiral switch based on the intermolecular ET from the UCNPs to the chiral switch. Since the UV emissions from the UCNPs have spectral overlap with the absorption bands of the open‐ring form, UV light from UCNPs upon 808 nm excitation can cause the photocyclization of the chiral switch from the colorless open‐ring to a colored closed‐ring form. Furthermore, the closed‐ring form can change back to the open‐ring form when exposed to the visible emission from UCNPs upon 980 nm excitation due to the spectral overlap between the visible emission and the absorption of the closed‐ring form (Figure 8c). As a result, the helical superstructure can display reversible handedness inversion upon irradiation by near‐infrared light of 808 or 980 nm (Figure 8d). The study describes a strategy to get responsive upconverting emission and handedness by regulating the ET between the UCNPs and chiral switch within a chiral structure.

#### 4.2.3 ET Regulated by Additives, Ultrasonics and Humidity

Compared to the small‐molecule N*LCs, responsive control of ET of lanthanide‐doped NPs in a chiral nematic structure built from biomass materials is rare, especially by electric field and light regulation. The CNCs are often used only as the matrix due to their high‐strength property, rather than the fantastic optical property originating from the chiral nematic structure. For example, Wang et al.^[^ ^122^ ^]^ achieved tunable color light emission by introducing LaPO_4_:Ln^3+^ NPs into the CNCs. Fedorov et al.^[^ ^120^, ^134^ ^]^ prepared up‐converting hybrid films based on the CNCs matrix doping with Sr_1‐_ *_x_* Ho*_x_* F_2+_ *_x_* particles. In such examples, however, CNCs were exploited only as a flexible, durable, transparent matrix. The chiral nematic structure effect on luminescence is far from being exploited. Current tuning approaches of PBG of CNCs mainly belong to passive methods, including adding additives, applying ultrasonics, or changing humidity.

**Figure 9:**
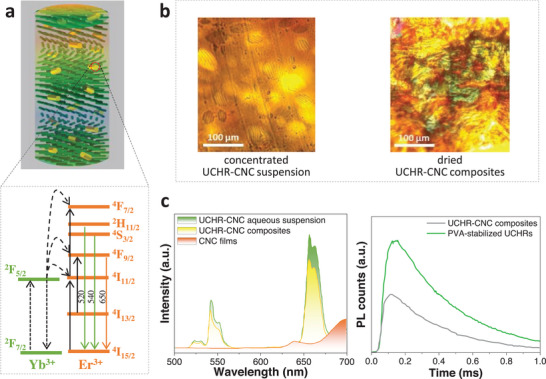
a) Schematic diagram showing the chiral upconverting films prepared through the co‐assembly of CNCs with PVA‐stabilized upconversion nanorods (UCHR) and the energy level scheme of Yb^3+^ and Er^3+^. b) POM images of concentrated (left) and dried nanorods‐CNC (right). c) Left: Upconversion emission of nanorods‐CNC aqueous suspension, nanorods‐CNC composites, and CNC films. Right: Lifetime of upconversion emission at 655.5 nm for nanorods‐CNC composites and PVA‐stabilized nanorods. Reproduced with permission.^[^ ^117^ ^]^ Copyright 2016, Wiley‐VCH.

As mentioned above in Section 4.1, Nguyen et al.^[^ ^117^ ^]^ prepared the first chiral upconverting films through co‐assembling CNCs with PVA‐stabilized NaYF_4_: 20 wt.% Yb^3+^, 2 wt.% Er^3+^ hexagonal nanorods (**Figure** **9**a). As the water evaporates, the chiral nematic structure forms with the appearance of striped tactoids in the aqueous suspension of nanorods‐CNC (Figure 9b, left). When the water had fully evaporated, fingerprint textures with uniform distance were observed, indicating the formation of the chiral nematic structure (Figure 9b, right). The resulting film exhibited strong upconversion emission when excited at 980 nm (Figure 9c, left). The effect of CNCs matrix on upconversion luminescence at 655.5 nm is also shown in Figure 9c, right. Both Zhao et al.^[^ ^135^ ^]^ and Morales‐Narváez et al.^[^ ^136^ ^]^ have reported that higher ET upconversion efficiency is observed in chiral NaYF_4_:Yb^3+^, Er^3+^/cellulose than the achiral materials, which is ascribed to the better dispersibility of UCNPs in photonic chiral ordered CNC films. However, a shorter lifetime was found for the chiral composites than the pristine nanorods. The authors explained this likely arises from the higher ET efficiency of upconversion nanorods in the chiral nematic CNCs. Following this pioneering work, tuning of PBG of 1D PCs prepared from CNCs and spontaneous emission/ET have been realized by methods based on additives, ultrasonics, and humidity.

#### 4.2.4 Tuning the PBG by Additives

**Figure 10:**
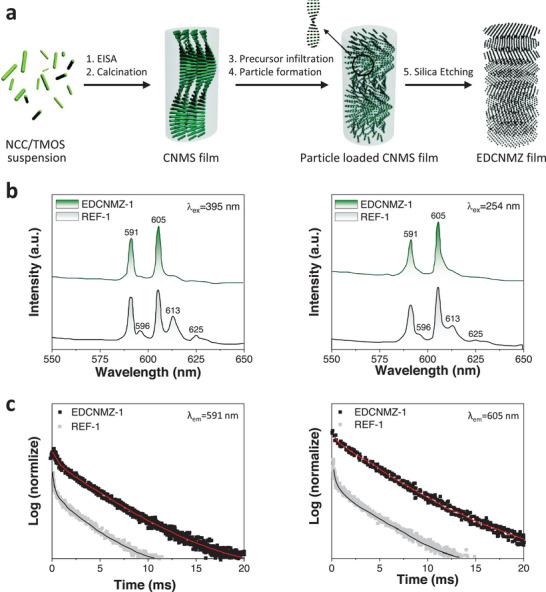
a) Schematic diagram showing the preparation of EDCNMZ‐n film. b) The emission spectra of EDCNMZ‐1 and REF‐1 excited by 395 nm (left) and 254 nm (right). c) The decay curves of EDCNMZ‐1 and REF‐1 monitored at 591 nm (left) and 605 nm (right) (*λ*_ex_ = 395 nm). Reproduced with permission.^[^ ^141a^ ^]^ Copyright 2014, Royal Society of Chemistry.

CNCs can be used as templates to synthesize mesoporous and chiral materials from silica,^[^ ^137^ ^]^ organosilica,^[^ ^138^ ^]^ titania,^[^ ^139^ ^]^ and polymers,^[^ ^140^ ^]^ among others.^[^ ^40^ ^]^ For example, mesoporous and chiral films composed of ZrO_2_:Eu^3+^ were prepared by CNC‐templated silica (**Figure** **10**a). By changing the ratio of tetramethyl orthosilicate to nanocrystalline cellulose, films with different PBG were prepared. Upon excitation, energy can transfer from the electron‐hole pairs of ZrO_2_ to Eu^3+^. Chiral nematic ordering of the resulting PCs EDCNMZ inhibits the ^5^D_0_ → ^7^F_1_ transition (596 nm) and the ^5^D_0_ → ^7^F_2_ transitions (613 and 625 nm) of Eu^3+^, while increasing the luminescence lifetime, compared to the reference sample without the chiral nematic structure (Figure 10 b,c).^[^ ^141^ ^]^ Similarly, the effect of chiral nematic structure on ET between O^2−^ and Eu^3+^ in Y_2_O_3_:Eu^3+^ was also studied by Chu et al.,^[^ ^142^ ^]^ where they observed that suppression of the ^5^D_0_ → ^7^F_2_ transition at 612 nm was accompanied with the increase of luminescence lifetime.

**Figure 11:**
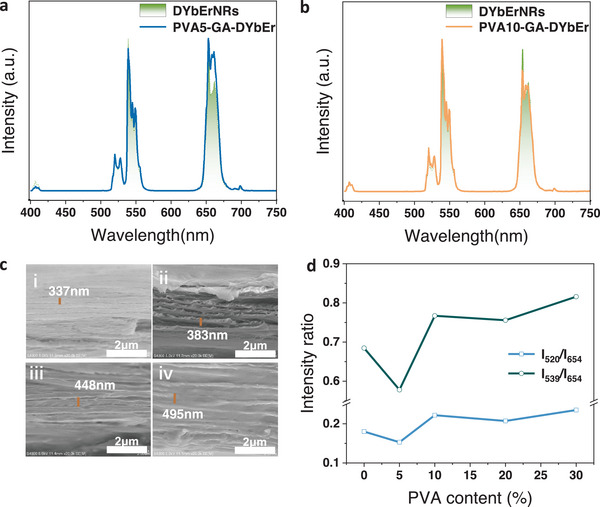
a) The upconversion luminescence spectra of DYbEr (dihydroxysuccinic acid modified NaGdF_4_:Yb^3+^, Er^3+^) nanorods and PVA5‐GA‐DYbEr film (The film was made from 5% PVA, glutaraldehyde and DYbEr nanorods); b) The upconversion luminescence spectra of DYbEr nanorods and PVA10‐GA‐DYbEr film; c) The SEM images of films with mass fraction of PVA is i) 5%, ii) 10%, iii) 20% iv) 30%; d) The intensity ratio of I_520_/I_654_ and I_539_/I_654_ with the increase of mass faction of PVA. Reproduced with permission. ^[^ ^143^ ^]^ Copyright 2022, Elsevier B.V.

The PBG of cellulose nanocrystal films can be tuned from UV to visible range by changing the amount of PVA. The film with tunable PBG exhibited the ability to modulate the downshifting luminescence and upconversion luminescence in our work, which can be enhanced by 28% and 18%, respectively. **Figure** **11**a‐b demonstrates the upconversion luminescence of DYbEr nanorods and PVA‐GA‐DYbEr films with PBG at 518 nm and 589 nm, which can be observed from the SEM images (Figure 11c). Figure 11d shows the intensity ratio of I_520_/I_654_ and I_539_/I_654_ signals as a function of polyvinyl alcohol content. Compared with the reference, the 520 nm and 539 nm emission decrease due to the PBG effect, while 654 nm emission increases because of the band edge effect in the PVA5‐GA‐DYbEr film with PBG at 518 nm. As for other films with PBG at 589, 688, and 759 nm, the 520 and 539 nm emissions were enhanced, while 654 nm emission intensity decreases due to the PBG effect, leading to the increase of value of I_520_/I_654_ and I_539_/I_654_.^[^ ^143^ ^]^

#### 4.2.5 Tuning the PBG by Ultrasonic Treatment

The PBG can also be tuned by ultrasonic treatment during the process of film preparation. Chu et al.^[^ ^142^, ^144^ ^]^ fabricated chiral nematic luminescent films by attaching YVO_4_:Eu^3+^ NPs to the surface of the twisted CNCs, where the CNCs self‐assembled as a chiral structure. The PBG of the chiral nematic films can be regulated by ultrasonic treatment, resulting in PBG centered at 342 nm, 472 nm, and 550 nm. The quantum yield rises with an increase in the PBG. The corresponding PBG also had an effect on the luminescence dynamics. The longest decay time of YVO_4_:Eu^3+^ NPs was achieved with the PBG at 472 nm, which overlapped with the excitation at 464 nm. The decay time was shorter when the PBG was at 342 or 550 nm, which had no overlap with the excitation spectrum.

**Figure 12:**
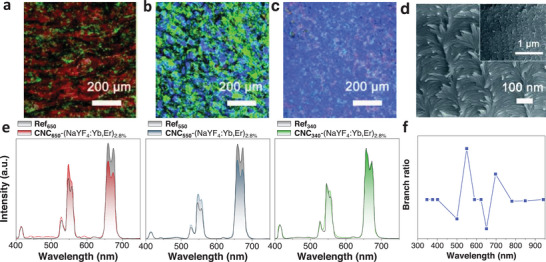
a–c) POM characterization of the films with PBG at 650, 550, and 340 nm (from left to right). d) SEM image of the film. e) The emission spectra of CNC_650_‐(NaYF_4_:Yb,Er)_2.8%_ (left), CNC_550_‐(NaYF_4_:Yb,Er)_2.8%_ (middle), and CNC_340_‐(NaYF_4_:Yb,Er)_2.8%_ (right). f) Intensity ratio of I(^4^F_9/2_ −^4^I_15/2_)/I((^2^H_11/2_, ^4^S_3/2_) – ^4^I_15/2_) as a function of PBG. Reproduced with permission.^[^ ^145^ ^]^ Copyright 2016, Royal Society of Chemistry.

Jiang et al.^[^ ^145^ ^]^ also prepared chiral composite films by the self‐assembly of CNCs and a series of NaYF_4_:Yb, Er. The optical property can be regulated by changing the PBG of the CNCs 1D PC through the ultrasonication method. The films with different PBG at 650, 550, and 340 nm, were prepared by ultrasonic treatment and designated as CNC_650_‐(NaYF_4_:Yb,Er)_2.8%_, CNC_550_‐(NaYF_4_:Yb,Er)_2.8%_ and CNC_340_‐(NaYF_4_:Yb,Er)_2.8%_, respectively. The POM images of these samples showed strong birefringence (**Figure** **12**a–c). An SEM image further verified the left‐handed chiral structure (Figure 12d). As for CNC_650_‐(NaYF_4_:Yb,Er)_2.8%_, the green emission from 520 nm to 570 nm increased, while the red emission from 630 nm to 680 nm decreased significantly with the comparison to the reference sample without chiral nematic ordering (Figure 12e, left). In the case of the PBG centered at 550 nm, the green emission was suppressed because of the inhibition of the band gap. The red emission was at the band edge and was thus enhanced (Figure 12e, middle). Emission of CNC_340_‐(NaYF_4_:Yb,Er)_2.8%_ was unaffected as the PBG of 340 nm is not in the red or green emission ranges (Figure 12e, right). Figure 12f summarized the relationship between the intensity ratio (I(^4^F_9/2_ – ^4^I_15/2_)/I((^2^H_11/2_, ^4^S_3/2_) −^4^I_15/2_)) and PBG. For PBG centered at 500 nm, the intensity ratio dropped sharply due to the enhanced green emission located at the edge of PBG. When the PBG matched well with the location of green emission, e.g., 550 nm, the green emission was significantly suppressed and hence the intensity ratio increased. The same principle was also applied to the lower intensity ratio when the PBG increased to 650 nm. This work demonstrated that the ET process in chiral composite films can be modulated by tuning the PBG which drives photon propagation.

#### 4.2.6 Tuning the PBG by Humidity

**Figure 13:**
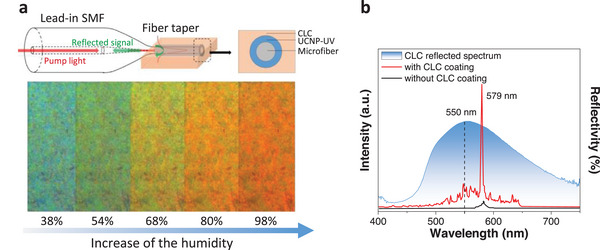
a) Top: Schematic illustration of the microfiber relative humidity sensor structure. Bottom: micrographs (magnification of 20×) of the planar CLC film at different relative humidity levels. b) Blue line: The CLC reflected spectrum. Red line: The emission of the fiber with CLC coating. Black line: The emission of the fiber without CLC coating. Reproduced with permission.^[^ ^146^ ^]^ Copyright 2019, Elsevier B.V.

A precise sensor for humidity based on UCNPs and cellulose liquid crystal (CLC) coating has been reported by Hu et al.^[^ ^146^ ^]^ The solid CLC film prepared by the self‐assembly of CNCs featured a left‐handed helical structure. Thus, the signal‐to‐noise ratio of the microfiber relative humidity sensor coated by UCNP‐diffused ultraviolet gel and CLC film can be enhanced due to the PBG effect of the CLC film (**Figure** **13**a **t**op). The UCNPs emission peak is located in the PBG of the film, which can be changed by absorbing or releasing H_2_O molecules. With increase in the relative humidity level, the emission was redshifted (Figure 13a bottom). The lasing emission of the microfiber sensor with CLC coating (red line) and without CLC coating (blue line) is displayed in Figure 13b. A narrower and stronger lasing signal centered at 579 nm is observed in the sample with CLC film, which is due to the band edge enhancement effect of the CLC film with the PBG of 550 nm (black dashed line in Figure 13b). In addition, the sensor has negligible temperature cross‐sensitivity. This study broadens the application of lanthanide‐doped NPs in a chiral nematic structure with better performance, e.g., higher accuracy and sensitivity.

#### 4.2.7 Examples of Regulating the CPL and their Application

**Figure 14:**
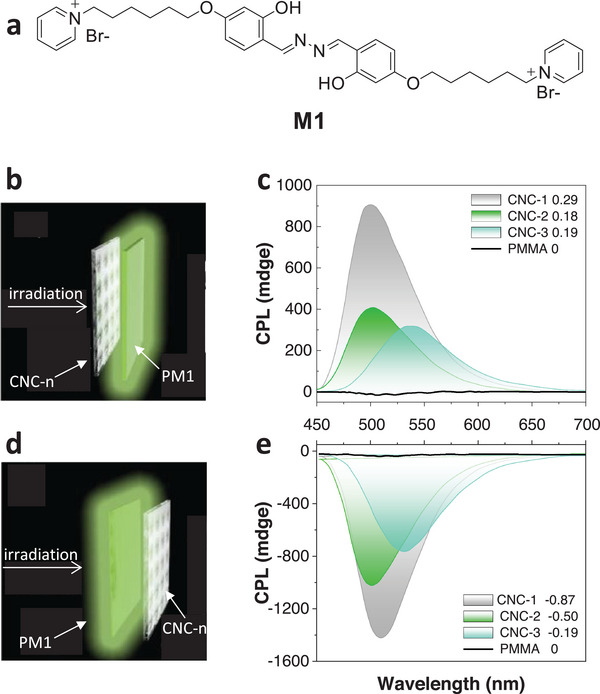
a) Chemical structure of the fluorescent dye M1. b) PM1 was put between CNC‐n and the CPL detector. c) The passive L‐CPL and *g*_lum_ values for each CNC‐n. d) PM1 was put front of the CNC‐n and the CPL detector. e) The passive R‐CPL and *g*_lum_ values for each CNC‐n. Reproduced with permission.^[^ ^150^ ^]^ Copyright 2018, Wiley‐VCH.

As mentioned above, one unique and important property of N*LCs is their ability to selectively reflect CPL. CPL was first observed by Samoilov^[^ ^147^ ^]^ on a chiral crystal of sodium uranyl acetate in 1948. The CPL of small molecules and polymeric materials has many potential applications in photonic devices, and therefore it has attracted immense attention. For example, the left‐handed chiral nematic structure produced by the self‐assembly of CNCs can reflect left‐handed CPL, leading to a positive circular dichroism signal for light transmission.^[^ ^148^ ^]^ A systematic study of CPL from Eu, Tb, Sm, Yb, Dy, and Cr complexes was provided by MacKenzie et al.^[^ ^149^ ^]^ for advanced security inks. The complexes obtained, through ingenious structural design, were characterized by maximized circularly polarized luminescence dissymmetry factor (*g*_lum_) and increased CPL properties. Zheng et al. ^[^ ^150^ ^]^ first demonstrated that CPL with extraordinary values of the *g*_lum_ can be generated and manipulated by the chiral structure of CNCs. By changing the PBG of the chiral cellulose encapsulated with luminophores, they realized CPL with a large *g*_lum_ value, tunable handedness, and wavelength. Due to the left‐handed structure, the composite chiral cellulose films can not only selectively reflect passive L‐CPL and transmit passive R‐CPL under incident light but also transform the spontaneous emission of luminophores into R‐CPL emission. This intrinsic ability of the chiral cellulose films was further verified by changing the relative position of the cellulose films with different PBG (CNC‐n) and the M1‐loaded poly(methyl methacrylate) film (briefed as PM1: M1 is shown in **Figure** **14**a). The L‐CPL emission of PM1 was reflected by the CNC‐n, then transmitted through PM1, and detected if PM1 was put between CNC‐n and the CPL detector (Figure 14 b and c). The R‐CPL emission of PM1 was detected when reversing positions of CNC‐n and PM1, resulting in the selective reflection of L‐CPL and transmission of R‐CPL (Figure 14 d and e). Furthermore, the work also illustrated the regulation of *g*_lum_ values by changing the PBG of CNC‐n.

**Figure 15:**
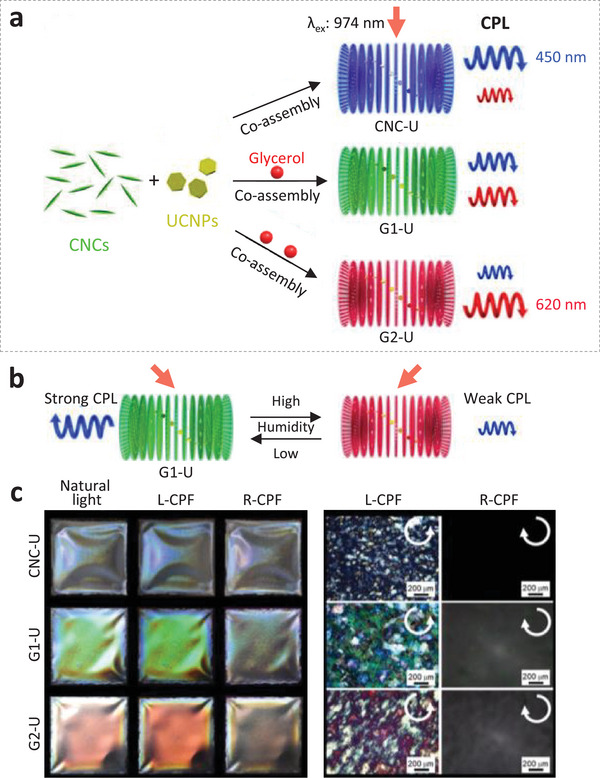
a) Schematic diagram showing the preparation of chiral photonic films with tunable UC‐CPL emissions through changes on PBG by the additive glycerol. b) Schematic diagram showing UC‐CPL emission of the glycerol‐containing photonic film G1‐U tuned by humidity. c) Left: Photographs of chiral photonic films taken under natural light, L‐CPF, and R‐CPF. Right: POM images of the films observed under L‐CPF and R‐CPF. Reproduced with permission.^[^ ^62c^ ^]^ Copyright 2019, American Chemical Society.

Li et al.^[^ ^62c^ ^]^ also prepared UCNPs‐CNCs chiral photonic films and achieved right‐handed, tunable UC CPL and *g*_lum_ by changing the PBG of CNCs. Rather than preparing chiral photonic films with different PBG by ultrasonic treatment, glycerol was used in this study as an external stimulus to tune the PBG. Films with PBG at 414 nm (0 g glycerol, referred to as CNC‐U), 509 nm (0.02 g glycerol, referred to as G1‐U), and 639 nm (0.04 g glycerol, referred to as G2‐U) were fabricated. **Figure** **15**a showed the well‐controlled UC‐CPL emission through changes in PBG of chiral photonic films by glycerol. As a result of the strong hygroscopicity of glycerol, the PBG of G1‐U was shown to be tuned by relative humidity, leading to a humidity‐responsive emission (Figure 15b). When the humidity increased from 33% to 85%, the peak maxima of PBG red‐shifted from 505 to 581 nm and the *g*_lum_ value decreased from 0.156 to 0.033. Circularly polarizing filters (CPF) were used to investigate the CPL of these photonic films (Figure 15c, left). The iridescent colors of all films observed under natural light and left‐handed CPF were brighter than that under right‐handed CPF. Furthermore, POM images showed the birefringence only under the L‐CPF due to the left‐handed chiral structures (Figure 15c, right). This work describes a simple and efficient approach to obtain humidity‐responsive UC‐CPL emission of a 1D chiral photonic crystal.

## 5 Summary and Outlook

LCs have the characteristics of both the flowability of liquid and the rigid order of crystals. Cholesteric LCs and nanocellulose LCs are chiral thermotropic and lyotropic LCs, respectively. Both of these are 1D photonic crystals. In this Review, we first discussed the effect of a photonic crystal on ET. Next, we focused on two kinds of 1D photonic crystals and summarized their formation and control methods of the chiral structure, followed by a survey on the effect of the structure on ET in lanthanide‐doped NPs.

Light and electric field control of the ET in lanthanide‐doped NPs in cholesteric LCs was then discussed. The cholesteric LC host acts as the photonic crystal and can respond to light and an electric field, leading to structural changes and tuning of the ET between lanthanides. The cholesteric LCs also exhibit characteristic luminescence themselves: for example, 5CB emits blue light when excited by 334 nm radiation. The emission from the LCs can be coupled with the lanthanide ions to realize a specific emission.

Compared with the cholesteric LCs research in ET, CNCs have been far from exploited. Cellulose and its derivatives are usually used as matrices for luminescent NPs, other than constituting a necessary functional part. Actually, the formed chiral nematic structure can have a great effect on the performance of materials due to the intrinsic flowability of liquid and the rigid order of crystals. CNCs LCs not only have unique properties due to their chiral structure but are also biocompatible materials that can be decomposed completely. The chiral nematic structure effect on optical properties has not yet been fully studied.

Thus, the self‐assembly conditions need to be precisely controlled to avoid defect formation and obtain a uniform chiral nematic structure. Research to look into the mechanism of the self‐assembly process of CNC is scarce. In addition, poor compatibility between the N*LCs and the dopants remains problematic. More effort should be input to maintain the chiral structure in the presence of a high concentration of dopants, such as luminescent NPs and polymeric additives.

More research is needed to explore the responsive control of ET between lanthanide‐activated NPs in biosourced CNC chiral nematic structures, especially stimulated by electrical and magnetic fields. As environmentally friendly hosts for lanthanide ions, CNCs can be used in many areas, for example, in the life science field. Except for the 1D PCs made by cholesteric LCs and self‐assembly of CNCs, 2D and 3D PCs can also be made by other forms of cellulose and its derivatives.^[^ ^151^ ^]^ There are also other responsive photonic crystals (RPCs), such as magnetic RPCs (Fe_3_O_4_), electrical RPCs (silica, polystyrene, Fe_3_O_4_@SiO_2_, ZnS@SiO_2_, elastomers, block copolymer photonic gels), mechanical RPCs, chemical RPCs, thermal RPCs, and optical RPCs.^[^ ^39^ ^]^ The effect of RPCs on the ET in lanthanide‐doped NPs remains enticing for future research and practical applications.

## Conflict of Interest

The authors declare no conflict of interest.
